# Ripening of Nonaqueous Emulsions of *n*‑Decane in Dimethyl Sulfoxide Observed by Time-Resolved Spin-Echo Small-Angle Neutron Scattering (SESANS)

**DOI:** 10.1021/acs.langmuir.4c05364

**Published:** 2025-05-19

**Authors:** E. Wouter Grünewald, Robert M. Dalgliesh, Steven R. Parnell, Wim G. Bouwman, Gregory N. Smith

**Affiliations:** † Reactor Institute, 2860 Technische Universiteit Delft, Mekelweg 15, 2629 JB Delft, The Netherlands; ‡ ISIS Neutron and Muon Source, Science and Technology Facilities Council, 120797 Rutherford Appleton Laboratory, Didcot OX11 0QX, United Kingdom

## Abstract

Since macroemulsions are only kinetically stable, characterizing their behavior as they change over time is relevant to their application. Time-of-flight spin-echo small-angle neutron scattering (SESANS) enables time-resolved measurement of the bulk evolution of concentrated, opaque emulsions without perturbing the system. Here, we present time-of-flight SESANS measurements of an *n*-decane-in-DMSO emulsion stabilized by Pluronic P-123, where changes in the system as it ripened were resolved. The radius of emulsion droplets were shown to grow over time with a rate of 25 μm^3^ h^–1^, suggesting that Ostwald ripening is the dominant aging process. Furthermore, SANS measurements revealed the presence of a stable population of swollen surfactant micelles, providing an additional mechanism for mass transfer between particles. Since time-of-flight SESANS can be used to obtain information about particle sizes, ripening rates, and associated processes, it is uniquely suited for studying the behavior of dense colloidal systems over time.

## Graphical abstract

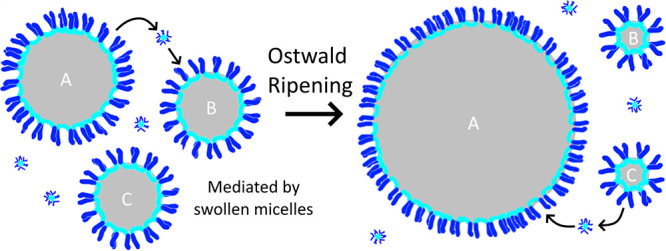

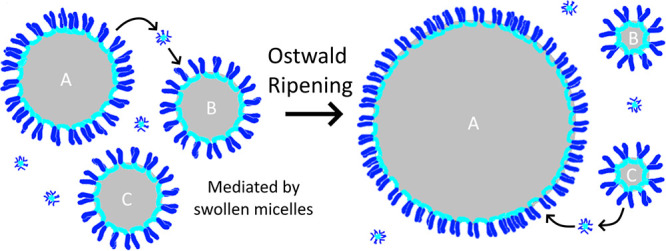

## Introduction

Emulsions are ubiquitous in science, industry, and daily life. Applications ranging from making dinner^1^ to ordered macroporous materials^2^ make the study of emulsion properties an important field. (Macro)­emulsions are only kinetically stable (as opposed to microemulsions, which are thermodynamically stable), and hence, the size of emulsion droplets will change over time.^3^ Droplets can flocculate, coalesce, or grow through a process known as Ostwald ripening.^4^ Emulsions, consisting of two immiscible liquids, often need to be stabilized by a third component. This emulsifier has a significant influence on the ripening behavior of the system. Commonly, surfactants are used for this, consisting of molecules with hydrophobic and hydrophilic (or in the case of nonaqueous emulsions, solvophobic and solvophilic) moieties. These molecules sit at the interface of the two liquids, stabilizing the emulsion droplets. The hydrophobic moiety often consists of a hydrocarbon or fluorocarbon chain containing 8–18 carbon atoms, while the hydrophilic part can be nonionic, ionic, or zwitterionic.^5^ Block or graft copolymers^6^ or proteins^7^ can also be used as surfactants to stabilize emulsions. These long molecules can give the emulsion properties difficult to achieve with simpler surfactants, for example, using proteins to create emulsions consisting of sticky droplets.^8^ Instead of surfactants, emulsions can also be stabilized by solid nanoparticles forming so-called Pickering emulsions.^9^

The nature and rate of the ripening process depends on the interplay between the two liquids and the emulsifier and, therefore, varies significantly between different systems. An important method to distinguish between different ripening processes is based on monitoring the droplet size over time. Ideally, such a method should be able to measure many droplets at once while they are in the bulk of a potentially concentrated emulsion. It should work over a wide range of droplet sizes, be noninvasive, and be quick enough to capture the kinetics of the system under study. Common methods used to size particles include (electron or light) microscopy, light scattering, ultrasonic spectrometry, and nuclear magnetic resonance (NMR).^1^ Microscopy often requires the emulsion to be diluted and placed on a microscope slide, thereby disturbing the system.^10^ Dynamic and static light scattering do not require this but are only possible when the sample is transparent to light, which limits the composition and concentration of emulsions that can be studied. Ultrasonic spectroscopy can be used to size concentrated emulsions *in situ*. However, it requires considerable knowledge of the thermophysical properties of the system, which may not be available for many relevant emulsions.^11^ NMR can also be used to obtain droplet size in concentrated emulsions, but the sample must be immobile for the duration of the measurement.^12^

Spin-echo small-angle neutron scattering (SESANS) enables the measurement of structures ranging from tens of nanometers to tens of micrometers.^13^ This is comparable to the length scales of droplets in many industrially relevant emulsions. Moreover, since the technique uses the scattering of neutrons rather than visible light, it allows the measurement of opaque, concentrated emulsions by varying the contrast of the solvents and solutes, which is not possible with techniques based on visible light, such as microscopy and light scattering, because the refractive index contrast is fixed. Since neutrons have a high penetration power, they sample a statistically large volume of the emulsion. This means SESANS can measure the bulk properties of emulsions *in situ*, while maintaining the ability to contrast match using deuterated materials and remaining noninvasive, as is characteristic of neutron methods. Using time-of-flight SESANS, kinetics of emulsion ripening can be studied since a broad range of length scales can be accessed simultaneously via the measurement of a spectrum of neutron wavelengths at the same time.

In this work, a model system consisting of 50% *n*-decane in dimethyl sulfoxide stabilized by block copolymer Pluronic surfactant was measured using SESANS. Nonaqueous (or oil-in-oil) emulsions such as these have significant benefits in applications such as pharmaceutical delivery and polymer synthesis.^14^ For many applications, it is important to know the droplet size distribution and how it changes over time. The size distributions obtained using SESANS were compared to optical microscopy to assess the differences between the two methods. From the change of the droplet size distribution over time, the ripening mechanism of the emulsion could be deduced. The same emulsion was also measured using conventional SANS to obtain information on the swollen micelles also present in the sample, which may contribute to the ripening process.

## Experimental Section

### Materials

Dimethyl sulfoxide (DMSO, 99.8%) and *n*-decane (99%) were purchased from Thermo Fisher Scientific. Triblock copolymer poly­(ethylene glycol)–*block*–poly­(propylene glycol)–*block*–poly­(ethylene glycol) (P-123, PEG:PPG:PEG 20:70:20) was purchased from Sigma–Aldrich. The deuterated *n*-decane-*d*_22_ (97% purity, 99% D) and DMSO-*d*_6_ (99% purity, 99% D) were purchased from Cambridge Isotope Laboratories and Sigma–Aldrich, respectively. All materials were used as supplied.

#### Nonaqueous Emulsions

The formulation of the emulsions was inspired by the work of Imhof and Pine.^10^ The emulsions consisted of *n*-decane in DMSO, stabilized by Pluronic P-123 surfactant. The hydrogenous and deuterated variants of the solvents were mixed to obtain the desired scattering length densities (ρ).

To prepare the emulsions, first an 18 wt % solution of P-123 in DMSO was made by melting the surfactant and placing it in a vial. DMSO was added by weight until a solution with the desired concentration was made. The solution was then heated to 80 °C until the surfactant was dissolved. 300 μg of this solution was placed into a separate vial using a Pasteur pipet and 300 μL of *n*-decane was added using volumetric pipets. The amount of added *n*-decane was confirmed by weighing the vial before and after addition. This resulted in an emulsion with an approximate volume fraction of *n*-decane of 0.5.

#### Emulsions for SANS and SESANS Measurements

For SESANS measurements, it is important to minimize the scattering from the surfactant. Therefore, the hydrogenous and deuterated DMSO were mixed such that the average scattering length density was close to that of P-123 (0.424 × 10^–6^ Å^–2^). For SESANS, the ratio of hydrogenous to deuterated *n*-decane was chosen such that the contrast was small enough so as to not depolarize the neutron beam completely, but large enough to give sufficient signal. For the SANS measurements, the surfactant was dissolved in pure deuterated DMSO and the *n*-decane was mixed such that the scattering length densities of the *n*-decane and DMSO were the same. This was done so the scattering signal would be dominated by the surfactant structures. The exact values are given in Table 1.

**Table 1:** Scattering Length Densities (ρ) of the Different Emulsions Made

sample	ρ_DMSO_ (× 10^–6^ Å^–2^)	ρ_*n*‑decane_ (× 10^–6^ Å^–2^)	Δρ (× 10^–6^ Å^–2^)
SESANS	0.453	0.798	0.345
SANS	5.28	5.17	0.11

The mixture was emulsified by shaking. The vial was shaken by hand for 20 s and then using a mechanical 25 Hz vortex shaker for 20 s. This was repeated three times, after which the emulsion was allowed to rest for a short time. The emulsion was then alternately mechanically shaken and allowed to rest until a very viscous emulsion was formed.

For the neutron scattering experiments, the emulsion was placed in a cylindrical quartz cuvette with a 1 mm path length using a syringe. To prevent creaming, the cuvettes were placed in a nonmagnetic rotating sample rack and slowly, continuously tumbled throughout the measurement.

In addition to the emulsions, a solution of 18 wt % P-123 in deuterated DMSO was prepared to measure using SANS.

### Methods

#### Optical Microscopy

The emulsion was imaged using a Zeiss Axioscope 7 optical microscope with 100× magnification provided by Diamond Light Source, Rutherford Appleton Laboratory. The emulsion was prepared as described above and allowed to ripen for the desired time. A small amount of liquid was then transferred to another vial and diluted by a factor of 4 in DMSO to be suitable for microscopy. A droplet of the diluted emulsion was placed on a glass slide and covered by a glass coverslip before being placed under the microscope. Images were taken 1 and 4 h after preparation.

To obtain size histograms, droplets were manually identified and marked. The Hough transform included in the Fiji ImageJ software^15^ was then used to count the marked droplets. A kernel smoothing function was used to obtain a probability density estimate for comparison with the particle size distributions obtained from SESANS.^16^

#### Spin-Echo Small-Angle Neutron Scattering (SESANS)

Spin-echo small-angle neutron scattering (SESANS) measurements were performed on the Larmor instrument at the ISIS Neutron and Muon Source (Rutherford Appleton Laboratory, United Kingdom).^17^ Larmor is a combined time-of-flight SANS and SESANS instrument using neutrons between 2.6 and 10 Å (polarized (SE)­SANS) or between 0.5 and 12.5 Å (unpolarized SANS). In SESANS mode, it uses adiabatic RF flippers to implement the shaped magnetic field regions necessary for this technique. For this experiment, the RF flippers were set to 2.0 MHz (corresponding to a magnetic field of 68.56 mT) and the angle of the precession regions with the beam axis was set to 30°. The spin-echo length (δ) is related to the wavelength (λ), magnetic field (*B*), and field angle (θ_0_) as follows:

1 $$\delta =\frac{c\lambda^{2}L\;\cot\left(\theta_{0}\right)B}{2\pi }$$

where *c* is the Larmor constant (*c* = 4.6368 × 10^14^ T^–1^ m^–2^) and *L* is the length of the precession regions (*L* = 1.2 m). The instrument was calibrated using a well-defined silicon grating to find the relation between the spin-echo length and poleshoe angle θ_0_ for a given magnetic field strength.

The SESANS data were modeled as a system of dense hard spheres with a range of sizes. To account for the effect of the particle size distribution on the scattering signal, the scattering in reciprocal space *I*(*q*) was calculated using the decoupling approximation:^18^

2 $$I\left(q\right)=\mathrm{scale}\;\frac{\phi }{V}P\left(q\right)\left(1+\beta \left(q\right)\left(S\left(q\right)-1\right)\right)$$

where ϕ is the volume fraction, *V* is the average particle volume, and β is given by

$$\beta \left(q\right)=\frac{|\left\langle F\left(q\right)\right\rangle {|}^{2}}{\left\langle \right|F\left(q\right){|}^{2}\rangle }$$

Since the emulsion had a volume fraction of approximately 50%, a Percus–Yevick structure factor *S*(*q*) was used.^19^ The scattering form factor from a sphere is given by^20^

3 $$P\left(q,R\right)\equiv F^{2}\left(q,R\right)={\left(3\frac{\sin\left(qR\right)-qR\;\cos\left(qR\right)}{{\left(qR\right)}^{3}}\right)}^{2}$$

where *q* is the scattering vector and *R* the droplet radius. Since the emulsion droplets are not uniform in size, the scattering intensity must be calculated as the integral over the size distribution^21^

4 $$P\left(q\right)=\frac{1}{V}\int f\left(x;\mu ,\sigma \right)F^{2}\left(q,x\right)\;dx$$

where *f*(*x*; μ, σ) is a particle size distribution and *V* the average particle volume. For emulsions, this is commonly described by a log-normal distribution.^22^ Such a distribution is a function of *x* where ln­(*x*) is normally distributed.

The normalized scattering correlation function can be calculated as a function of the spin-echo length δ by

5 $$\frac{\ln\left(P/P_{0}\right)}{\lambda^{2}t}=\left(G\left(\delta \right)-G\left(0\right)\right)$$

where λ is the neutron wavelength, *t* the thickness of the sample, and *G*(δ) the Hankel transform^23^ of *I*(*q*):

6 $$G\left(\delta \right)=\frac{1}{2\pi }\int_{0}^{\infty }J_{0}\left(q\delta \right)I\left(q\right)q\;dq$$

7 $$G\left(0\right)={\left(\Delta \rho \right)}^{2}\phi \left(1-\phi \right)\xi$$

Δρ is the difference in scattering length density between the droplets and continuous phase, ϕ the volume fraction, and ξ the correlation length of the system.

SESANS data were reduced using custom scripts in the Mantid software package^24^ and modeled as described above using the SasView analysis software.^25^

#### Small-Angle Neutron Scattering (SANS)

Small-angle neutron scattering (SANS) measurements were performed on the Larmor instrument at the ISIS Neutron and Muon Source (Rutherford Appleton Laboratory, United Kingdom).^17^ With neutron wavelengths between 0.5 and 12.5 Å and a sample-to-detector distance of 4 m, a *q* range of 0.005 to 0.7 Å^–1^ was accessible. Data were converted from raw data to reduced data of scattering intensity, as a function of *q*, by correcting for the detector efficiency and sample transmission using Mantid.^24^ The data were placed on an absolute scale (cm^–1^) by measuring the scattering of a mixture of hydrogenous and deuterated polystyrene with known radius of gyration and scattering cross section.^26^

The polymer micelles visible in SANS can be modeled in terms of the self-correlation of the cores, the self-correlation term of the brushes, the cross term between the cores and chains, and the cross term between the different chains:^27^

8 $$I_{\mathrm{mic}}=N_{\mathrm{agg}}^{2}\beta_{\mathrm{core}}^{2}P_{\mathrm{core}}\left(q\right)+N_{\mathrm{agg}}\beta^{2}P_{\mathrm{brush}}\left(q\right)+2N_{\mathrm{agg}}^{2}\beta_{\mathrm{core}}\beta_{\mathrm{brush}}S_{\mathrm{brush}-\mathrm{core}}\left(q\right)+N_{\mathrm{agg}}\left(N_{\mathrm{agg}}-1\right)\beta_{\mathrm{brush}}^{2}S_{\mathrm{brush}-\mathrm{brush}}\left(q\right)$$

where

$$\begin{array}{l} \beta_{\mathrm{brush}}=V_{\mathrm{brush}}\left(\rho_{\mathrm{brush}}-\rho_{\mathrm{solv}}\right)\\ \beta_{\mathrm{core}}=\frac{V_{\mathrm{core}}\left(1-x_{\mathrm{solv},\mathrm{core}}\right)}{N_{\mathrm{agg}}}\left(\rho_{\mathrm{core}}-\rho_{\mathrm{solv}}\right)\\ P_{\mathrm{core}}\left(q,R_{\mathrm{core}}\right)=\Phi^{2}\left(qR_{\mathrm{core}}\right)\\ \Phi \left(qR\right)=3\frac{\sin\left(qR\right)-qR\;\cos\left(qR\right)}{{\left(qR\right)}^{3}}\\ P_{\mathrm{brush}}\left(q,R_{g}\right)=2\frac{\exp\left(-x\right)-1+x}{x^{2}},\mathrm{with}\;x=R_{g}^{2}q^{2}\\ S_{\mathrm{brush-core}}\left(q,R_{\mathrm{core}},R_{g},d\right)=\Phi \left(q,R_{\mathrm{core}}\right)\psi \left(qR_{g}\right)\frac{\sin\left(q\left(R_{\mathrm{core}}+dR_{g}\right)\right)}{q\left(R_{\mathrm{core}}+dR_{g}\right)}\\ \psi \left(qR_{g}\right)=\frac{1-\exp\left(-x\right)}{x}\\ S_{\mathrm{brush-brush}}\left(q,R_{\mathrm{core}},d,R_{g}\right)=\psi^{2}\left(qR_{g}\right){\left(\frac{\sin\left(q\left(R_{\mathrm{core}}+dR_{g}\right)\right)}{q\left(R_{\mathrm{core}}+dR_{g}\right)}\right)}^{2} \end{array}$$

The parameters of the implemented model are the radius of the micellar core *R*_core_, the grafting density (copolymer molecules *N*_agg_ per unit surface *S*) *n*_agg_, the molecular volume of the part of the polymer forming the corona *V*_brush_, the radius of gyration of the block unit in the corona *R*_*g*_, and the scattering length densities of the core, brush, and solvent (calculated to be 0.34, 0.64, and 5.28 × 10^–6^ Å^–2^, respectively). *x*_solv,core_ is the amount of solvent in the core, and *d* is the nonpenetration of the chains into the core, i.e., to what extent the parts of the polymer forming the corona of the micelle do not appear inside the core. It is assumed the solvophilic and solvophobic parts of the surfactant molecule are sufficiently separated and, hence, *d* = 1. The micelles were assumed to be monodisperse in the absence of *n*-decane and to have a log-normally distributed *R*_core_ when *n*-decane was present. A hard-sphere Percus–Yevick structure factor was also applied with an effective radius *R*_HS_ and volume fraction ϕ.

SANS data were modeled as described above using the SASfit analysis software.^28^

## Results and Discussion

Here, the droplet size distributions obtained using optical microscopy and SESANS are presented and compared. The microscopy shows considerably smaller sizes with a larger spread, especially at shorter times after emulsion preparation. Both show droplets increasing in size as the emulsion ages. Additionally, data obtained using SANS indicates a stable population of swollen surfactant micelles, which could play a role in the ripening mechanism.

### Microscopy

Microscopy images of the prepared emulsion were taken at different times. At later times, the droplets are clearly larger than at earlier times (Figure 1).

**Figure 1:**
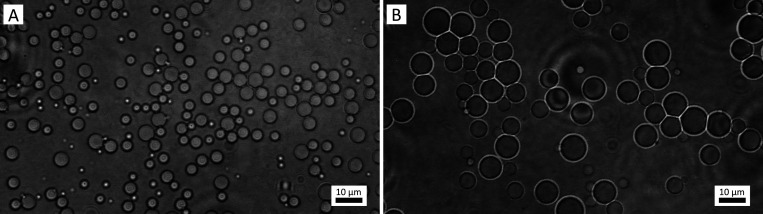
Optical microscopy images of an *n*-decane-in-DMSO emulsion stabilized by Pluronic P-123 either 1 h (A) or 4 h (B) after preparation. The emulsion was diluted by adding DMSO before being placed on the microscopy slide. The average droplet size increases with time after preparation.

Histograms were made for the size distribution after 1 and 4 h (Figure 2). A clear increase in mean size is visible, and while the emulsion after 4 h has a droplet size distribution akin to a Gaussian, droplets in the younger emulsion follow a bimodal distribution. It should be noted that obtaining quantitative data from these images in a systematic way is challenging, since the low resolution and large number of imaging artifacts make it difficult to accurately identify and size droplets. Furthermore, the distribution of sizes was not uniform over the sample and the manual analysis process limits the number of particles that can be considered. It was therefore interesting to investigate the emulsion with SESANS, being a method that can measure a large volume simultaneously and does not require the dilution and sample manipulation associated with microscopy.

**Figure 2:**
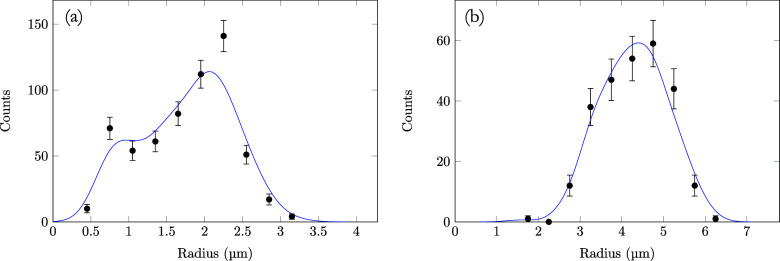
Histograms of droplet sizes obtained from the microscopy images after (a) 1 h and (b) 4 h. Poisson statistics were assumed for the number of droplets.

### Time-Resolved SESANS Measurements

In contrast to microscopy, SESANS can be used to study the emulsion in bulk without the need for dilution or other physical manipulation. In Figure 3, the SESANS results for measurements at three different time points are plotted. The measurement time for each curve was 10 min. The emulsion was measured more frequently at earlier time points to be able to resolve the faster change in droplet radius during that period. The sample was kept on the instrument for the entire duration of the experiment. The data are fitted with a polydisperse sphere model, where the size distribution is given by a log-normal distribution. To account for the correlation between droplets, a hard-sphere structure factor was used with the decoupling approximation.^18^ The normalized scattering correlation function plateaus at a larger spin-echo length at later times, indicating an increase in mean droplet size.^29^ Additionally, the correlation function plateaus at a more negative value due to the larger volume of the droplets (assuming contrast and volume fraction remain constant). The oscillatory features are due to the structure factor and indicate the droplet size distribution is relatively narrow. The fitted size distributions are plotted in Figure 4, with the distributions obtained by microscopy in Figure 2. The droplet sizes obtained by SESANS were larger and their distribution narrower than those measured using microscopy. This may be due to the additional manipulation involved in preparing the sample for microscopy, but it should also be noted that SESANS is more sensitive to larger particle sizes, and hence could skew the size distribution to larger values. Additionally, the fact that SESANS measures the bulk droplet sizes in situ also affects the resulting sizes and size distributions. The SESANS size distributions are narrower than measured for other emulsions^10,30^ and even narrower than the steady-state distribution from LSW theory.^31,32^

**Figure 3:**
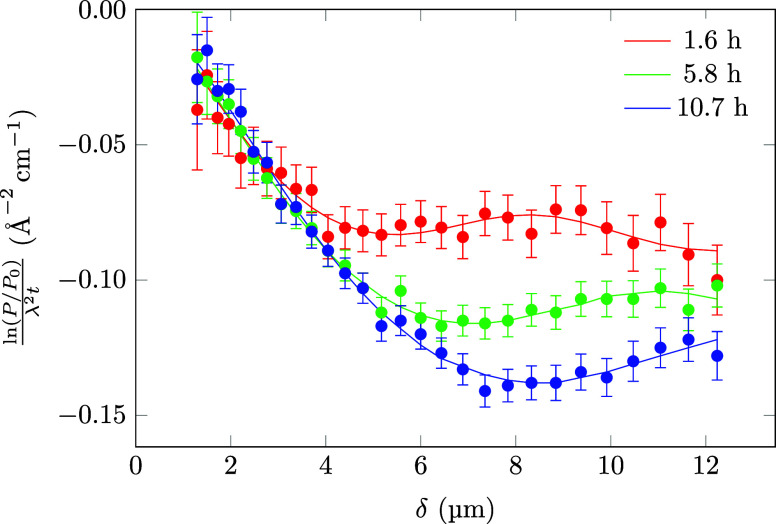
Normalized scattering correlation functions obtained using SESANS 1.6, 5.8, and 10.7 h after preparation. The data were fitted in SasView using polydisperse spheres with a hard-sphere structure factor. The decoupling approximation was used to describe the effects of the polydispersity on the structure factor.

**Figure 4:**
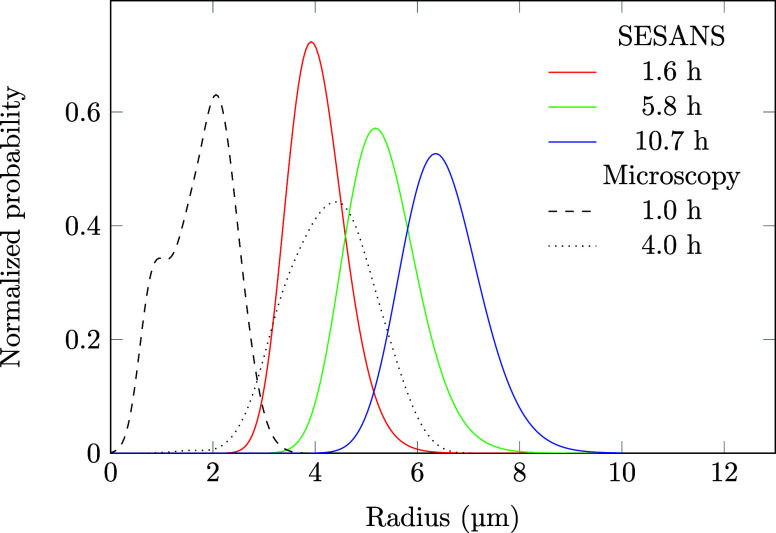
Log-normal radius distributions obtained from fitting concentrated polydisperse spheres to the SESANS curves in Figure 3. Distributions obtained from optical microscopy are added for comparison. All curves are scaled such that the area underneath is unity.

### SANS

While SESANS was used to measure emulsion droplets with sizes on the order of 1 to 10 μm, time-of-flight SANS was used to investigate the presence and kinetics of smaller structures in the emulsion. First, a solution of 18 wt % P-123 in DMSO-*d*_6_ was measured which confirmed the presence of spherical surfactant micelles (Figure 5). For the SANS measurement of the emulsion, deuterated and hydrogenous *n*-decane were mixed such that the scattering length densities of the *n*-decane and DMSO-*d*_6_ were close to equal. This means the scattering signal is dominated by surfactant micelles, except at low values of *q* where the emulsion droplets become dominant. In the emulsion, micelles are also present but in a swollen state compared to pure P-123/DMSO-*d*_6_. This is indicated in the SANS curve by a shift of the peak to a lower value of *q*. The swelling is likely due to solubilization of *n*-decane inside the core of the micelles. Swollen micelles are an important mass transfer mechanism^32^ and can greatly influence the ripening rate. Figure 6 shows how the SANS signal from the emulsions changes over time. The peak coming from the swollen micelles does not change, and therefore the size and number of micelles remained constant. Meanwhile, the intensity of the slope at low *q* decreased over time, which in the Porod limit indicates a decrease in specific droplet surface area. P-123 is a nonionic surfactant, so the micelles can be desorbed from the surface of the emulsion and adsorbed on the surface of another, thereby moving *n*-decane from one droplet to another and enhancing the rate of Ostwald ripening.^33^ Since the SANS data show that the concentration of micelles remains constant, the adsorption and desorption processes are balanced on the time scale of the measurement.

**Figure 5:**
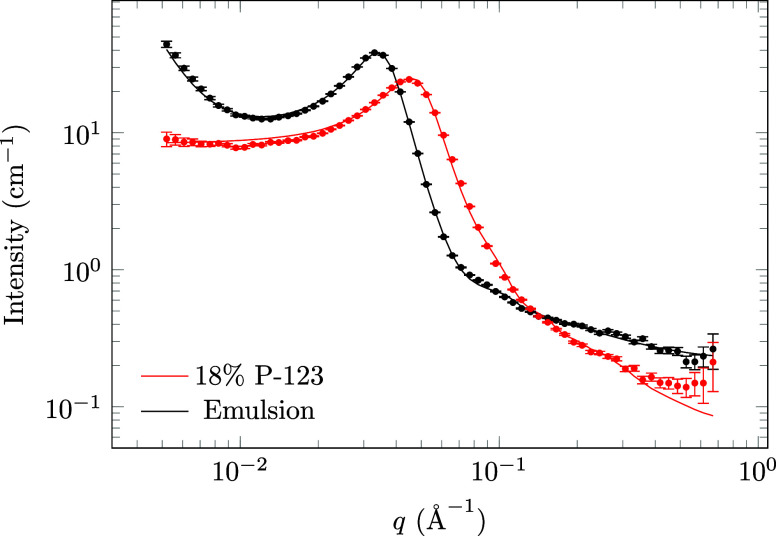
SANS data from a *n*-decane-in-DMSO emulsion, compared to that of 18 wt % P-123 micelles. The patterns were fitted with a spherical polymer micelle model and a flat background in SASfit.^28^ For the emulsion, a *q* ^–4^ power law from the emulsion droplets and a background from deuterated *n*-decane in hydrogenous *n*-decane were also taken into account. The shift of the peak to lower *q* indicates an increase of the micelle size, likely due to the micelles being swollen with *n*-decane.

**Figure 6:**
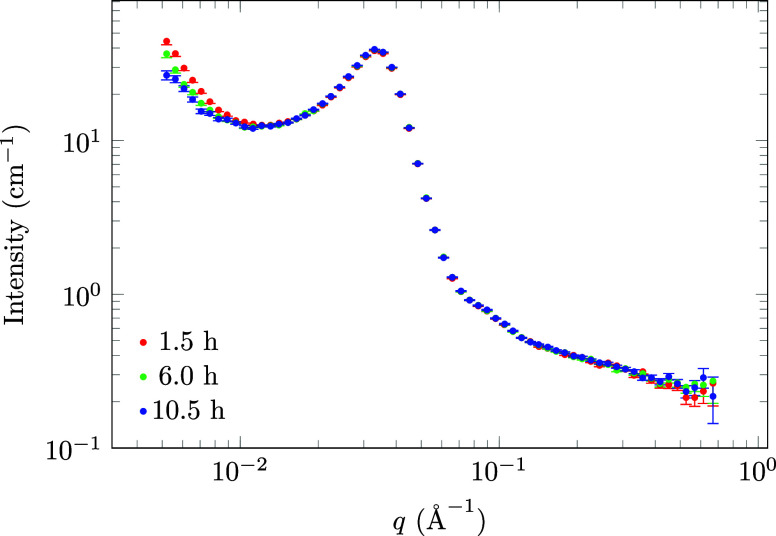
Emulsion SANS measurements taken at different ages of the emulsion. The peak from the swollen micelles stays the same, while the slope at low *q* becomes less steep. In the Porod limit, this indicates a decrease in specific area (i.e., droplet surface per unit volume) and, hence, an increase in the mean droplet radius.

Emulsion droplets can grow due to a number of different processes. Flocculation causes droplets to agglomerate, forming larger particles made from multiple individual droplets. If the surfactant film between the droplets is then ruptured, they merge into a large droplet. Ostwald ripening, however, is a process in which molecules of the dispersed phase diffuse from smaller droplets to large ones due to a difference in chemical potential. Each of these processes will lead to a different change of the mean droplet radius *R̅* over time. If the rate-limiting step is the collision of individual droplets, as can be the case for flocculation and coalescence, *R̅* ^3^ will grow linearly in time.^34^ If during coalescence the film rupture is limiting, 1/*R̅* or 1/*R̅* ^2^ changes linearly in time.^1^ Ostwald ripening also causes a linear change of *R̅* ^3^.^31^ It is therefore interesting to look at the change of the mean droplet radius over time to deduce the mechanism of droplet growth. As can be seen in Figure 7, SESANS shows that *R̅* ^3^ increases linearly in time. Since these emulsions are highly concentrated, the droplets are continuously touching. This makes it unlikely that droplet collisions would be the rate-limiting step. Furthermore, Imhof and Pine^10^ have shown that the ripening can be halted by adding a small amount of silicone oil, indicating that Ostwald ripening is likely to be the dominant process in this emulsion. The ripening rate determined using SESANS is 25 μm^3^ h^–1^, much higher than the Ostwald ripening rate calculated from Lifshitz–Slyozov–Wagner (LSW) theory (1.73 μm^3^ h^–1^).^31,35^ This theory of Ostwald ripening assumes an infinitely dilute emulsion in surfactant-free conditions, so it is not unexpected for this concentrated, surfactant-stabilized emulsion to deviate. Increasing the concentration decreases the distance between droplets, thereby making it easier for *n*-decane molecules to diffuse from one droplet to another. Additionally, the SANS data presented above indicates the presence of a stable population of swollen surfactant micelles, providing an additional mechanism for mass transfer.^33^ The exact role of micelles in the mass transfer between dispersed phase droplets in Ostwald ripening, and their importance relative to molecular diffusion, is a debated topic. Above the critical micelle concentration, an increase in the surfactant concentration has been reported to increase the ripening rate,^36,37^ which is also seen here. A number of different mechanisms of for the role of micelles in mass transport between droplets have been suggested.^32,33,38^ If micelles can approach the surface of an emulsion droplet, exchange of dispersed phase molecules can take place due to collision of micelles with the surface. Swollen micelles may also desorb from one droplet surface and collide with another. A third mechanism is the uptake into micelles of dissolved dispersed phase molecules from the continuous medium. These mechanisms provide additional pathways for molecules to move from one droplet to another and therefore increase the ripening rate.

**Figure 7:**
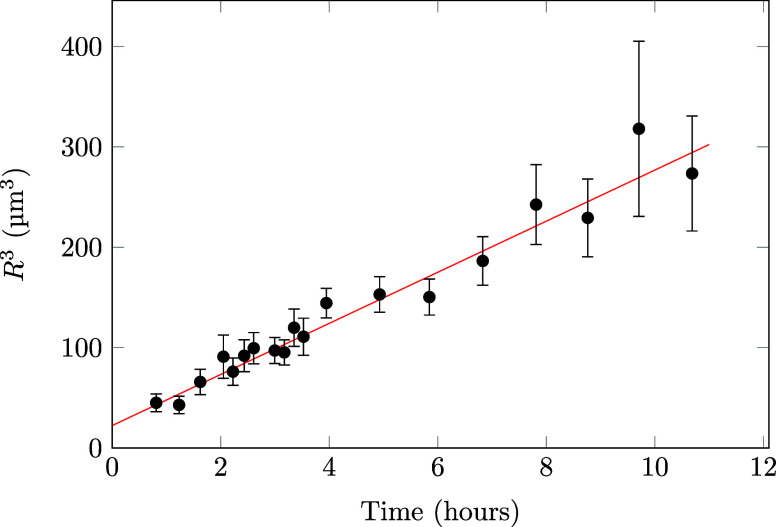
Cube of the mean droplet radius obtained from fitting concentrated polydisperse spheres to the SESANS data is plotted against time. The ripening rate of the droplets is 25 μm^3^ h^–1^ (±2, χ^2^ = 0.50).

## Conclusions

Measurements using time-of-flight SESANS to study the kinetic behavior of a concentrated *n*-decane-in-DMSO emulsion are presented. The cube of the mean radius increases linearly in time, consistent with the theory of Ostwald ripening. A faster ripening rate compared to LSW theory can be explained by a combination of the high volume fraction of emulsion droplets and the presence of swollen surfactant micelles, observed using SANS, mediating the mass transfer process. It is therefore insufficient to only consider the change in droplet size in an emulsion system with a high concentration of surfactant—smaller micellar structures play an important role in the ripening process and behavior of the system. Many current methods cannot study the bulk behavior of both droplets and micelles without considerably altering the system. By combining SANS and SESANS, a more robust understanding of these complex systems can be obtained. When comparing the size distributions measured using SESANS and those obtained from microscopy, significant differences are seen. This may be a difference between the bulk properties of the concentrated system and that prepared for microscopy. While the microscopy measurements required the emulsion to be diluted and placed between glass slides, SESANS was able to measure the kinetics of droplets in the bulk system without significant sample manipulation.

To further explore the mechanism of ripening in concentrated emulsions and the role of surfactant micelles therein, additional SESANS experiments could be performed. While we were able to determine the rate of Ostwald ripening in the system, the relative importance of different mechanisms and parameters, such as the concentration of emulsion droplets, the influence of coalescence, and the presence and concentration of micelles, could not be distinguished. By reducing the concentration of surfactant and comparing the ripening rate, the role of micelles in the ripening process can be further investigated. Adding an insoluble species, such as silicone oil as used by Imhof and Pine,^10^ is expected to stop Ostwald ripening and allow investigation of other ripening processes present in this system. Finally, the ratio of *n*-decane to DMSO could be varied to study the influence of droplet concentration. A comprehensive set of experiments would be able to decouple the parameters influencing the ripening process and give insight into the relative importance of different mechanisms.

## Supplementary Material



## Data Availability

Data are available in ref 17.

## Notes

Modeled SESANS data using the droplet size distribution measured by microscopy, a duplicate measurement and SANS fitting parameters (PDF)

## ¶

For E. Wouter Grünewald: PSI Center for Neutron and Muon Sciences, Forschungsstrasse 111, 5232 Villigen PSI, Switzerland

## §

For S. R. Parnell: ISIS Neutron and Muon Source, Science and Technology Facilities Council, Rutherford Appleton Laboratory, Didcot, OX11 0QX, United Kingdom.

## References

[1] McClements, D. J. Food Emulsions, 2nd Edition; CRC Press, 2004.

[2] Imhof A., Pine D. J. (1997). Ordered macroporous
materials by emulsion templating. Nature.

[3] Eastoe, J. In Colloid Science: Principles, Methods and Applications, 2nd Edition; Cosgrove, T. , Ed.; John Wiley, 2010; Chapter 5, pp 91–115.

[4] Vincent, B. In Colloid Science: Principles, Methods and Applications, 2nd Edition; Cosgrove, T. , Ed.; John Wiley, 2010; Chapter 6, pp 117–133.

[5] Tadros, T. F. An Introduction to Surfactants, 1st Edition; De Gruyter: Berlin, 2014.

[6] Tadros T. (2006). Principles
of emulsion stabilization with special reference to polymeric surfactants. J. Cosmet. Sci..

[7] McClements D. J. (2004). Protein-stabilized
emulsions. Curr. Opin. Colloid Interface Sci..

[8] Hoffmann H., Reger M. (2014). Emulsions with unique
properties from proteins as emulsifiers. Adv.
Colloid Interface Sci..

[9] Chevalier Y., Bolzinger M.-A. (2013). Emulsions stabilized with solid nanoparticles:
Pickering
emulsions. Colloids Surf., A.

[10] Imhof A., Pine D. J. (1997). Stability of Nonaqueous
Emulsions. J. Colloid Interface Sci..

[11] Coupland, J. N. , McClements, D. J. In Food Emulsions, 4th Edition; Friberg, S. , Larsson, K. , Sjoblom, J. , Eds.; CRC Press, 2004; Chapter 14, pp 573–593.

[12] Ling N. N., Haber A., May E. F., Fridjonsson E. O., Johns M. L. (2017). By-line NMR emulsion droplet sizing. Chem. Eng. Sci..

[13] Bouwman W. G. (2021). Spin-echo
small-angle neutron scattering for multiscale structure analysis of
food materials. Food Struct..

[14] Zia A., Pentzer E., Thickett S., Kempe K. (2020). Advances and opportunities
of oil-in-oil emulsions. ACS Appl. Mater. Interfaces.

[15] Schindelin J., Arganda-Carreras I., Frise E., Kaynig V., Longair M., Pietzsch T., Preibisch S., Rueden C., Saalfeld S., Schmid B. (2012). Fiji: an open-source platform for biological-image
analysis. Nat. Methods.

[16] Bowman, A. W. ; Azzalini, A. Applied Smoothing Techniques for Data Analysis: The Kernel Approach with S-Plus Illustrations, Vol. 18; OUP Oxford, 1997.

[17] Bouwman, W. ; Smith, G. ; Grünewald, W. ; Parnell, S. Evolution of droplet size in nonaqueous emulsions using time-resolved, time-of-flight SESANS. ISIS Neutron Muon Source Data J. 2022, 10.5286/ISIS.E.RB2220716.

[18] Kotlarchyk M., Chen S. (1983). Analysis of small angle neutron scattering
spectra from polydisperse
interacting colloids. J. Chem. Phys..

[19] Ashcroft N. W., Lekner J. (1966). Structure and Resistivity
of Liquid Metals. Phys. Rev..

[20] Guinier, A. ; Fournet, G. Small-angle Scattering of X-Rays; John Wiley, 1955.

[21] King, S. ; Kienzle, P. Polydispersity and Orientational Distributions. 2015. Available via the Internet at: https://www.sasview.org/docs/user/qtgui/Perspectives/Fitting/pd/polydispersity.html (accessed March 30, 2023).

[22] Boxall J. A., Koh C. A., Sloan E. D., Sum A. K., Wu D. T. (2010). Measurement
and calibration of droplet size distributions in water-in-oil emulsions
by particle video microscope and a focused beam reflectance method. Ind. Eng. Chem. Res..

[23] Bakker J. H., Washington A. L., Parnell S. R., van Well A. A., Pappas C., Bouwman W. G. (2020). Analysis of SESANS data by numerical Hankel transform
implementation in SasView. J. Neutron Res..

[24] Arnold O. (2014). Mantid–Data analysis
and visualization package for neutron
scattering and *μ* SR experiments. Nucl. Instrum. Methods Phys. Res., Sect. A.

[25] SasView for Small Angle Scattering Analysis. 2023; https://www.sasview.org (accessed March 30, 2023).

[26] Wignall G. D., Bates F. S. (1987). Absolute calibration
of small-angle neutron scattering
data. J. Appl. Crystallogr..

[27] Pedersen J. S., Gerstenberg M. C. (1996). Scattering
form factor of block copolymer micelles. Macromolecules.

[28] Kohlbrecher J., Breßler I. (2022). Updates in
SASfit for fitting analytical expressions
and numerical models to small-angle scattering patterns. J. Appl. Crystallogr..

[29] Andersson R., van Heijkamp L. F., de Schepper I. M., Bouwman W. (2008). Analysis of spin-echo
small-angle neutron scattering. J. Appl. Crystallogr..

[30] Maaref S., Ayatollahi S. (2018). The effect
of brine salinity on water-in-oil emulsion
stability through droplet size distribution analysis: A case study. J. Dispersion Sci. Technol..

[31] Wagner C. (1961). Theorie der
Alterung von Niederschlägen durch Umlösen (Ostwald-reifung). Z. Elektrochem..

[32] Koroleva M. Y., Yurtov E. V. (2021). Ostwald ripening in macro-and nanoemulsions. Russ. Chem. Rev..

[33] Weiss J., Coupland J. N., Brathwaite D., McClements D. J. (1997). Influence
of molecular structure of hydrocarbon emulsion droplets on their solubilization
in nonionic surfactant micelles. Colloids Surf.,
A.

[34] Evans, D. F. ; Wennerstrom, H. The Colloidal Domain; VHC Publishers, 1996.

[35] Sakai T., Kamogawa K., Nishiyama K., Sakai H., Abe M. (2002). Molecular
diffusion of oil/water emulsions in surfactant-free conditions. Langmuir.

[36] Djerdjev A. M., Beattie J. K. (2008). Enhancement of Ostwald Ripening by Depletion Flocculation. Langmuir.

[37] Binks B., Clint J., Fletcher P., Rippon S., Lubetkin S., Mulqueen P. (1999). Kinetics of swelling of oil-in-water emulsions stabilized
by different surfactants. Langmuir.

[38] Kabalnov A. S. (1994). Can micelles
mediate a mass transfer between oil droplets?. Langmuir.

