# Supplementary material for: Ripening of Nonaqueous Emulsions of n‑Decane in Dimethyl Sulfoxide Observed by Time-Resolved Spin-Echo Small-Angle Neutron Scattering (SESANS)
Source: Langmuir. 2025 May 19;41(21):12883–9. doi: 10.1021/acs.langmuir.4c05364 (PMC12139035; doi:10.1021/acs.langmuir.4c05364)
Supplement: Supplementary file 1 [file la4c05364_si_001.pdf]

Supporting Information—Ripening of nonaqueous emulsions of n-decane in dimethyl sulfoxide observed by time-resolved spin-echo small-angle neutron scattering (SESANS)

E. Wouter Grünewald,[†,¶] Robert M. Dalgliesh,[‡] Steven R. Parnell,[†,§] Wim G. Bouwman,[†] and Gregory N. Smith[*,‡]

†*Technische Universiteit Delft, Reactor Institute, Mekelweg 15, 2629 JB Delft, The Netherlands*

‡*ISIS Neutron and Muon Source, Science and Technology Facilities Council, Rutherford Appleton Laboratory, Didcot, OX11 0QX, United Kingdom*

¶*Current address: PSI Center for Neutron and Muon Sciences, Forschungsstrasse 111, 5232 Villigen PSI, Switzerland*

§*Current address: ISIS Neutron and Muon Source, Science and Technology Facilities Council, Rutherford Appleton Laboratory, Didcot, OX11 0QX, United Kingdom*

E-mail: gregory.smith@stfc.ac.uk

SESANS fitted with bimodal distribution

The size distributions obtained by microscopy and SESANS presented in the main text are rather different in both shape and mean size. It is interesting to consider what the SESANS signal of a bimodal distribution such as the one obtained by microscopy on the 1 hour old emulsion would look like. In figure S1, SESANS data is fitted both with the method described in the main paper, and using the size distribution obtained by microscopy. A scale factor was applied to the SESANS model for fitting in both cases. As can be seen, a reasonable fit can be achieved in both cases (within the statistical uncertainty of the data). The width of the microscopy distribution smooths out the oscillations due to the structure factor. The fact that two size distributions which are so different both give reasonable fits is due to the limitations of SESANS: its sensitivity to larger particles and difficulty to reliably estimate the shape of the particle distribution.

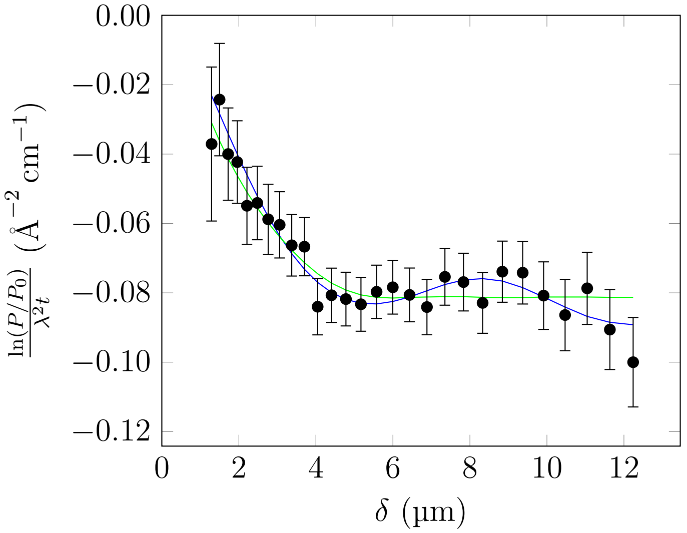

Figure S1: SESANS data fitted with the same curve as in the main paper using a lognormal size distribution (blue), and fitted using the bimodal distribution obtained by microscopy using a scale factor as fitting parameter.

SANS fitting parameters

Table S1: Parameters of the `SPHERE+chains` model. Numbers parentheses were estimated or calculated, all others were simultaneously fitted.
* Fitted with a log-normal distribution with $\bar{R} = 15.9$ Å and $\sigma = 0.31$.

	Surfactant micelles			Emulsion
	5%	10%	18%	
ϕ	0.058	0.173	0.276	0.302
R_{HS}(Å)	62.0			85.5
R_{core}(Å)	6.6			*
$x_{solv,core}$	(0)			(0.95)
n_{agg}(Å^{-2})	0.061			
V_{brush}(Å^3)	(1463)			
R_g(Å)	20.8			

Duplicate SESANS measurement

A duplicate sample was measured to assess the reproducability of the method. A plot of R^3 against time is given in figure S2. The ripening rate of this duplicate sample is the same as for the initial sample within their statistical significance.

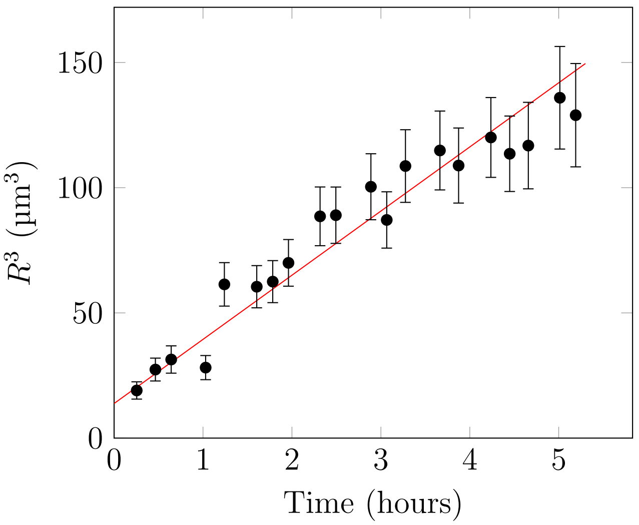

Figure S2: The growth behaviour of the duplicate sample. The cube of the mean droplet radius obtained from fitting concentrated polydisperse spheres to the SESANS data is plotted against time. The ripening rate of the droplets is 25.62 μm^3 h^{-1} (±1.47, $\chi^2 = 1.01$).
